# Bilateral Laparoscopic Partial Nephrectomies: A Case Report

**DOI:** 10.1089/cren.2015.0007

**Published:** 2015-11-01

**Authors:** Sravan Panuganti, Simpa Samuel Salami, Louis Raphael Kavoussi

**Affiliations:** ^1^ Department of Urological Surgery, Rowan University School of Osteopathic Medicine, Stratford, New Jersey.; ^2^ Department of Urology, University of Michigan Medical School, Ann Arbor, Michigan.; ^3^ The Arthur Smith Institute for Urology, Hofstra North Shore-LIJ School of Medicine, New Hyde Park, New York.

## Abstract

Although laparoscopy is a recognized operative approach to the management of renal masses, there is currently no standardized approach to manage bilateral synchronous renal masses. We present a case of synchronous bilateral renal masses, identified during work-up for flank pain, and managed simultaneously with laparoscopic partial nephrectomies. The patient is a 42-year-old Caucasian male found to have bilateral renal masses during evaluation for left flank pain. Cross-sectional imaging studies showed a 7.0 × 7.3 × 5.2 cm anterior, mid-to-lower pole mass on the left kidney and a 1.5 × 1.9 × 1.6 cm medial lower pole mass on the right kidney. He underwent bilateral laparoscopic partial nephrectomy at the same setting, with an uncomplicated postoperative course. Pathology report revealed clear cell renal-cell carcinoma (ccRCC) on both sides. He had normal renal function and no evidence of recurrence in the first 6 months of follow-up. This case demonstrates the possibility and safety of performing bilateral laparoscopic partial nephrectomies in one operative session. Our review of the literature supports the role of genetic counseling and the need for long-term surveillance in young patients having RCC.

## Introduction and Background

Majority of renal masses are identified incidentally. Multiple tumors identified less than 6 months apart are classified as synchronous, whereas tumors identified more than 6 months apart are classified as metachronous. With only few cases reported in the literature, there is currently no consensus regarding the operative approach to the management of bilateral synchronous renal masses. Although it is agreed that a minimally invasive technique results in improved short-term outcomes, namely shorter hospital stay, decreased narcotic pain medication requirements, and short convalescence, there is no consensus on how to approach synchronous bilateral renal masses. Should they be staged? Which lesion first? Should the larger or the more technically difficult or both lesions be managed at the same setting? Should an open or minimally invasive approach be utilized? For the first time, to our knowledge, we present a case of bilateral laparoscopic partial nephrectomies performed in a single operative session.

## Case Presentation

**FIG. 1.**
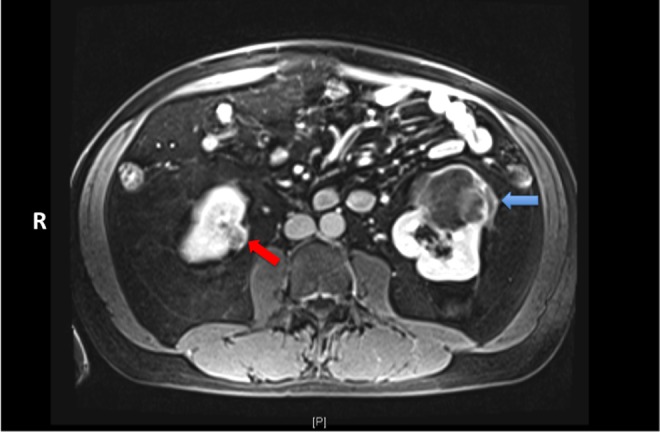
Axial cut of a contrast-enhanced MRI showing a 7.0 × 7.3 × 5.2 cm anterior, mid-to-lower pole left renal mass (*blue arrow*) and a 1.5 × 1.9 × 1.6 cm medial lower pole right renal mass (*red arrow*).

The patient is a 42-year-old Caucasian male found to have bilateral renal masses incidentally during work-up for left flank pain caused by an obstructing ureteral stone. After an uneventful ureteroscopy and laser lithotripsy of the left 7 mm ureteral calculus at an outside hospital (OSH), he was referred to our institution 1 month later for evaluation of his renal masses. He was asymptomatic at this time. His medical history was significant for diabetes mellitus type 2 and hyperlipidemia (both controlled with diet and exercise), as well as hypertension managed with losartan. He denied any family history of genitourinary or other malignancies. Physical examination was unremarkable. His laboratory tests, including complete blood count, basic metabolic profile, and liver function tests, were within normal limits. A noncontrast abdominal CT performed at the OSH showed a 7.0 × 7.3 × 5.2 cm anterior, mid-to-lower pole mass on the left kidney, and a 1.5 × 1.9 × 1.6 cm medial lower pole mass on the right kidney. An MRI with contrast confirmed both renal masses with enhancement concerning for malignancy (Fig. 1). Chest X-ray showed no evidence of metastatic disease. The patient underwent an uneventful bilateral laparoscopic partial nephrectomy. The operation was performed using the standard laparoscopic technique in the supine position, and thus, there was no need to reposition, prepare, or drape the patient after the first side was done. The patient was secured on the operative table and we air planed the table toward the contralateral side to facilitate bowel mobilization. The smaller right-sided mass was excised first without clamping the renal vessels and the larger left-sided renal mass was excised next using a hilar vascular clamp for 14 minutes (estimated blood loss = 1200 mL). The pathology report of both renal masses showed clear cell renal-cell carcinoma (ccRCC; right, pT1b Fuhrman grade 2; left, PT1a Fuhrman grade 1) with negative margins. The patient was discharged from the hospital on postoperative day 3. At 6-month follow-up, his serum creatinine was 0.97 mg/dL and he had no evidence of recurrence.

## Discussion and Literature Review

Although bilateral laparoscopic or hand-assisted renal surgeries have been described, there is no report, to our knowledge, of bilateral laparoscopic partial nephrectomies performed in a single setting.^1,2^ There is currently no consensus on how to approach synchronous bilateral renal masses being considered for partial nephrectomies.

In this case, we approached the smaller lesion first because it appeared to be less technically challenging. We were able to extirpate the tumor without clamping the renal vascular supply. Since this was effective, we were able to proceed to the larger lesion, which was more technically challenging and required clamping of the renal hilar vasculature. Our goal was to avoid clamping the hilar vessels of both renal units, which may result in acute kidney injury necessitating a transient need for dialysis. From an oncologic perspective, however, some authors may argue that the larger mass should be addressed first. The argument is that if it became unsafe to proceed with the operation, the higher risk lesion would have been addressed and the small lesion could be removed at a future time point or even kept under observation. However, if one were to clamp the vessels supplying the kidney with the larger mass, then the patient almost definitely is committed to a staged operation. Furthermore, an experienced laparoscopist performed this operation effectively without clamping one of the renal units. It is possible that the use of a robot-assisted technique may be necessary in less-experienced hands.

Paired organs are susceptible to the same genetic and environmental carcinogenic influences. It is thought that as many as 3% to 5% of patients having unilateral RCC will develop bilateral disease in their lifetime.^3^ Those with genetic predisposition for developing bilateral RCC have risk factors such as a young age at primary diagnosis, a multifocal primary tumor, immediate family history of RCC, hereditary diseases such as von Hippel Lindau, Birt-Hogg-Dubé, hereditary papillary renal carcinoma, familial clear cell renal carcinoma, or hereditary leiomyomatosis RCC.^3^ Wiklund et al.^3^ reported that age less than 60 years at diagnosis was associated with a 90% increased risk of developing bilateral RCC, whereas those younger than 40 years at diagnosis had an 1800% increased risk of bilateral disease compared with the general population. Our patient was 42 years old at diagnosis and was properly counseled, but he is yet to undergo genetic testing.

It is unclear whether synchronous or metachronous disease at a young age portends a less favorable prognosis. In those having metachronous disease, a longer time interval between incidence of the first and second tumor led to a more favorable prognosis.^4^ The second tumor was often at a higher TNM stage and Fuhrman grade than the primary tumor. Regardless of which histologic subtype of RCC, the class has been documented to be the same in bilateral kidney tumors in over 95% of bilateral RCCs.^5^ In our case, both lesions were ccRCC subtype. Approximately two-thirds of cases of all RCCs are clear cell in nature and this frequency appears to be maintained in bilateral disease.^5^ This understanding may be useful in cases being considered for surveillance, especially when the histology analysis result of one of the lesions is known.

Since RCC tends to be passed down through generations and early age of onset may be a sign of hereditary RCC, we agree with the recommendations of Shuch et al. that all patients having RCC at 46 years or younger undergo genetic counseling and germline mutation testing for the disease.^6^ The recommendation was based on a retrospective cohort analysis of the SEER-17 cancer database, where an age cutoff of 46 years maximizes sensitivity (70%) and specificity (90%) while limiting the number needed to test lies at the 10th percentile of age of diagnosis for all patients having RCC in the database. The recommended age allowed 7 to 38 patients to be tested for identifying one case of hereditary RCC and this will account for detecting 70% of all hereditary cases.^6^ Our patient was appropriately counseled to undergo genetic testing.

The current American Urological Association guidelines recommend screening up to 5 years after surgery in moderate- to high-risk patients treated for RCC, those who have pT2-4 disease. However, as many as half of metachronous tumors can be found past 5 years after the initial nephrectomy.^4^ Hence, we counseled our patient and planned to put him on surveillance for up to 10 years.

## Conclusion

In conclusion, simultaneous laparoscopic bilateral partial nephrectomies are possible in experienced hands. Although it is unclear which lesion to approach first (the smaller or the larger), the goal should be oncologic control, preservation of renal function, and subjecting the patient to as few operations as possible. Long-term follow-up, genetic counseling, and testing should be considered in patients having RCC at a young age.

## Abbreviations Used

ccRCC: clear cell renal-cell carcinoma
CT: computed tomography
MRI: magnetic resonance imaging
TNM: TNM Classification of Malignant Tumors
OSH: outside hospital
